# Highly Efficient and Selective Photocatalytic Nonoxidative Coupling of Methane to Ethylene over Pd-Zn Synergistic Catalytic Sites

**DOI:** 10.34133/2022/9831340

**Published:** 2022-11-07

**Authors:** Yanduo Liu, Yihong Chen, Wenbin Jiang, Tingting Kong, Pedro H. C. Camargo, Chao Gao, Yujie Xiong

**Affiliations:** ^1^School of Chemistry and Materials Science, University of Science and Technology of China, Hefei, Anhui 230026, China; ^2^Institute of Energy Hefei Comprehensive National Science Center, Hefei, Anhui 230031, China; ^3^Key Laboratory of Functional Molecular Solids, Ministry of Education, Anhui Engineering Research Center of Carbon Neutrality, College of Chemistry and Materials Science, Anhui Normal University, Wuhu, Anhui 241000, China; ^4^Department of Chemistry, University of Helsinki, FIN-00014, Finland

## Abstract

Photocatalytic nonoxidative coupling of CH_4_ to multicarbon (C_2+_) hydrocarbons (e.g., C_2_H_4_) and H_2_ under ambient conditions provides a promising energy-conserving approach for utilization of carbon resource. However, as the methyl intermediates prefer to undergo self-coupling to produce ethane, it is a challenging task to control the selective conversion of CH_4_ to higher value-added C_2_H_4_. Herein, we adopt a synergistic catalysis strategy by integrating Pd-Zn active sites on visible light-responsive defective WO_3_ nanosheets for synergizing the adsorption, activation, and dehydrogenation processes in CH_4_ to C_2_H_4_ conversion. Benefiting from the synergy, our model catalyst achieves a remarkable C_2+_ compounds yield of 31.85 *μ*mol·g^−1^·h^−1^ with an exceptionally high C_2_H_4_ selectivity of 75.3% and a stoichiometric H_2_ evolution. In situ spectroscopic studies reveal that the Zn sites promote the adsorption and activation of CH_4_ molecules to generate methyl and methoxy intermediates with the assistance of lattice oxygen, while the Pd sites facilitate the dehydrogenation of methoxy to methylene radicals for producing C_2_H_4_ and suppress overoxidation. This work demonstrates a strategy for designing efficient photocatalysts toward selective coupling of CH_4_ to higher value-added chemicals and highlights the importance of synergistic active sites to the synergy of key steps in catalytic reactions.

## 1. Introduction

Under the reality of insufficient coal and oil stockpiles, conversion of methane (CH_4_), which is the predominant component in natural gas, biogas, shale gas, and combustible ice, to value-added chemical feedstocks is an intriguing approach for sustainable development [1–4]. However, as a nonpolar molecule with tetrahedral symmetry, CH_4_ has a high C-H bond energy which requires high energy input (i.e., high operating temperatures and pressures) to cleave the C-H bond [5–7]. Additionally, such hash reaction conditions commonly lead to the production of undesired but thermodynamically favorable overoxidized products (i.e., CO and CO_2_) [8–11]. Given such circumstance, there are giant economic and environmental incentives for developing efficient sustainable approaches to achieve selective CH_4_ conversion toward the target products.

Photocatalysis, employing inexhaustible solar energy instead of thermal energy, provides an attractive alternative route to sustainable CH_4_ conversion under ambient reaction conditions [11–15]. Among various methane conversion schemes, nonoxidative coupling of CH_4_ to ethylene (C_2_H_4_) along with simultaneous production of H_2_ is a preferable pathway, as C_2_H_4_ is the high value-added key chemical feedstock, while H_2_ is an important clean energy carrier. Nevertheless, it is still a grand challenge to achieve efficient and selective conversion of CH_4_ to C_2_H_4_, mainly because photocatalysts often lack efficient active sites for activation of C-H bond, and the generated methyl radicals upon activation prefer to undergo self-coupling toward production of less valued ethane (C_2_H_6_) [16–20]. In this regard, there is an urgent need to rationally engineer active sites on the photocatalyst surface for synergizing the adsorption, activation, and dehydrogenation processes to enable in achieving the efficient and selective photocatalytic nonoxidative coupling of CH_4_ to C_2_H_4_.

Among various reported active sites, the Zn^+^−O^−^ pairs in ZnO have been well recognized as efficient active sites for photocatalytic CH_4_ activation and coupling, and as such, ZnO has been extensively applied to construct photocatalysts for CH_4_ conversion [21–23]. However, due to the wide band gap, ZnO can only absorb ultraviolet light, which severely restricts their catalytic efficiency in practical application under sunlight. In addition, the insufficient dehydrogenation capability for Zn^+^ sites limits the efficiency of C_2_H_4_ production. To achieve the goal of CH_4_ to C_2_H_4_ nonoxidative coupling conversion, it is greatly desired, yet challenging, to implement the Zn^+^−O^−^ pairs in other photosensitive semiconductor materials with the light harvesting capacity in broad spectral range and to simultaneously introduce another active site with strong dehydrogenation capability.

Herein, we aim to adopt a synergistic catalysis strategy by integrating multiple active sites on a visible light-responsive substrate for harnessing the adsorption, activation, and dehydrogenation processes to achieve highly efficient and selective CH_4_ to C_2_H_4_ conversion. Taking the visible light-responsive defective WO_3_ as a model substrate, Zn^+^−O^−^ paired active sites are implemented into WO_3_ nanosheets with large specific surface area and suitable energy band position through a doping method [24, 25]. Furthermore, considering the strong dehydrogenation capability toward C-H bond, Pd sites are introduced on WO_3_ nanosheets by depositing Pd nanoparticles via a self-reduction process. Benefiting from the synergy of two active sites, our model catalyst achieves remarkable activity and selectivity for CH_4_ to C_2_H_4_ conversion as well as a nearly stoichiometric H_2_ evolution, benchmarked against the state-of-the-art photocatalysts. In situ spectroscopic studies reveal that the Zn sites promote the adsorption and activation of CH_4_ molecules, while the Pd sites facilitate the dehydrogenation of methoxy intermediates and suppress the overoxidation [26–28]. Moreover, we unravel the reaction pathway for CH_4_ to C_2_H_4_, in which the adsorbed CH_4_ is activated and dehydrogenated to in turn generate methyl, methoxy, and methylene intermediates, and finally, the methylene radicals undergo self-coupling reactions to produce C_2_H_4_. This work provides a new perspective for designing the photocatalyst through leveraging synergistic active sites and highlights the key role of strong dehydrogenation capability in enhancing the selectivity for CH_4_ to C_2_H_4_ conversion.

## 2. Results

**Figure 1:**
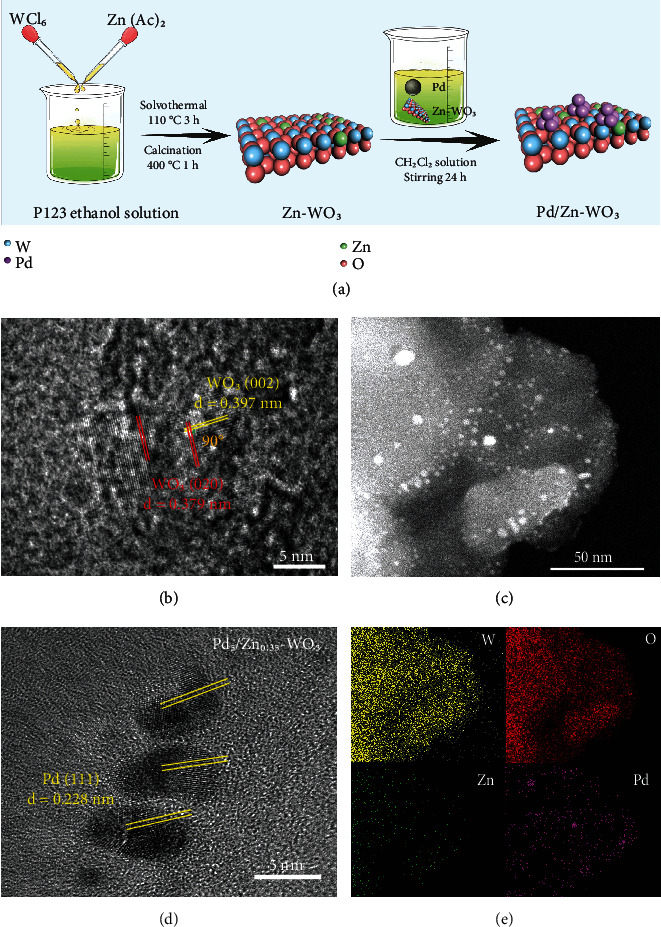
Schematic synthetic process and morphology characterization. (a) Schematic illustration for the synthetic protocol of the Pd-Zn comodified WO_3_ nanosheets. (b) HRTEM image of WO_3_. (c) HRTEM and (d) HAADF-STEM images of Pd_5_/Zn_0.35_-WO_3_. (e) The corresponding EDS elemental mapping of W (yellow), O (red), Zn (green), and Pd (purple) for Pd_5_/Zn_0.35_-WO_3_.

As illustrated in Figure 1(a), the Pd-Zn comodified WO_3_ nanosheets are constructed through a two-step protocol, in which Zn is in situ doped during hydrothermal synthesis of defective WO_3_ nanosheets, and subsequently, Pd nanoparticles are integrated by self-reduction. The prepared model catalyst is denoted as Pd_5_/Zn_0.35_-WO_3_ (5% and 0.35% refer to the theoretical mass fractions of Pd and Zn in the composite). The actual mass fractions of Pd and Zn determined by ICP-MS results are 4.46% (10.27 mg/L) and 0.31% (0.71 mg/L) in Pd_5_/Zn_0.35_-WO_3_ (230 mg/L). The x-ray diffraction (XRD) patterns (Figure S1a) show that the prepared defective WO_3_ substrate is monoclinic phase (JCPDS Card No. 83-0950), and the crystalline phase structure remains unchanged after Zn doping and Pd modification [25]. Such a result is consistent with that from UV–vis diffuse reflectance spectra (DRS, Figure S1b), showing that the samples maintain the inherent light absorption characteristics of defective WO_3_ substrate after the Zn doping and Pd modification. Taken together, the results indicate that the Pd-Zn comodification strategy has no negative effect on the basic physical properties of WO_3_ substrate.

Scanning electron microscopy (SEM) images (Figure S2a and S2b) reveal that the bare WO_3_ substrate has a nanosheet structure with a diameter of approximately 400 nm. Furthermore, transmission electron microscopy (TEM) also demonstrates the nanosheet morphology of the prepared Pd-Zn comodified WO_3_ (Pd_5_/Zn_0.35_-WO_3_). The clear lattice fringes with the spacings of 0.379 nm and 0.397 nm at an angle of 90° in a high-resolution TEM (HRTEM) images can be assigned to the (020) and (002) crystal planes of monoclinic phase WO_3_ (Figure 1(b)), which are consistent with the XRD results [25]. Meanwhile, such a nanosheet morphology remains unchanged after Zn doping and Pd modification (Figure S2c and S2d). Additionally, the aberration-corrected high-angle annular dark-field scanning TEM (HAADF-STEM) image of the Pd-Zn comodified WO_3_ nanosheets (Figure 1(c)) shows that Pd nanoparticles with an average diameter of 5 nm are highly dispersed on the WO_3_ substrate surface. The lattice fringes with an interplanar distance of 0.228 nm ascribed to the (111) plane of Pd nanoparticles are clearly observed in HRTEM image (Figure 1(d)) [28]. The corresponding energy-dispersive x-ray spectroscopy (EDS) elemental mapping (Figure 1(e)) demonstrates that Zn dopants and Pd nanoparticles are uniformly distributed on the surface of the WO_3_ substrate. Moreover, x-ray photoelectron spectroscopy (XPS, Figure S3) reveals that isolated Zn^2+^ ions have been successfully doped into the WO_3_ lattice while the zero-valent state of Pd proves that Pd nanoparticles are indeed formed by the self-reduction method. Note that low-valence W^5+^ species is resolved along with the binding energy of 531.8 eV for adsorbed oxygen species at the defects in the WO_3_ substrate by XPS, manifesting the existence of oxygen vacancies as defects [29, 30]. Taken together, the characterizations confirm that Zn sites and Pd nanoparticles have been successfully introduced onto WO_3_ substrate, constructing the Pd-Zn comodified WO_3_ nanosheets.

**Figure 2:**
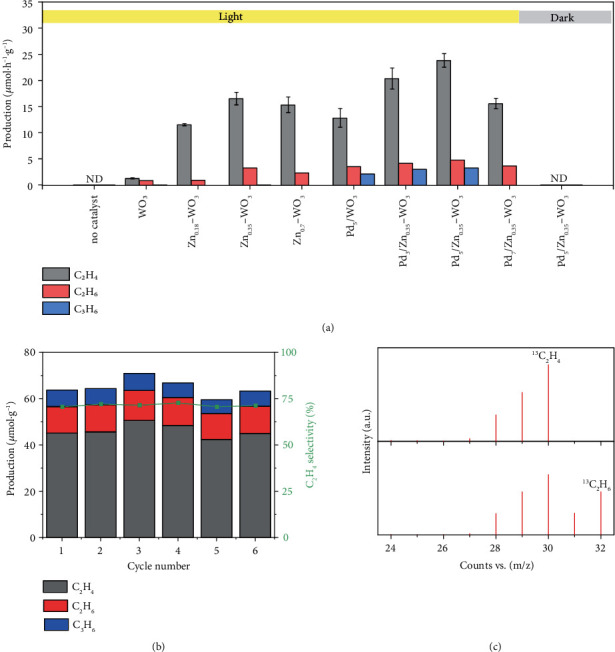
Photocatalytic performance for CH_4_ conversion. (a) C_2+_ compound yields for CH_4_ conversion by WO_3_, Zn_y_-WO_3_, Pd_x_/WO_3_, and Pd_x_/Zn_y_-WO_3_ (x% and y% refer to the mass fraction of Pd and Zn in the composite) photocatalysts under light irradiation of 2 h. “ND” stands for “not detected.” (b) Production rates of C_2_H_4_, C_2_H_6_, and C_3_H_6_, as well as the C_2_H_4_ selectivity in C_2+_ products, for photocatalytic CH_4_ coupling by Pd_5_/Zn_0.35_-WO_3_ in six successive cycling tests. (c) GC-MS data of ^13^C_2_H_6_ and ^13^C_2_H_4_ produced from photocatalytic ^13^CH_4_ coupling by Pd_5_/Zn_0.35_-WO_3_.

After confirming the formation of Pd-Zn comodified WO_3_ nanosheets, we then evaluate their performance as a photocatalyst for CH_4_ conversion under full-spectrum illumination. The catalytic performance of the synthesized Pd-Zn comodified WO_3_ (denoted as Pd_x_/Zn_y_-WO_3_, x%, and y% refer to the mass fractions of Pd and Zn in the composite) in reference to control samples are summarized in Figure 2(a). The CH_4_ coupling products of the optimized Pd_5_/Zn_0.35_-WO_3_ are C_2_H_6_, C_2_H_4_, and C_3_H_6_ (Figure S4, S5), in which the C_2+_ compounds yield reaches 31.85 *μ*mol·g^−1^·h^−1^ with a C_2_H_4_ selectivity of 75.3% (57% in total carbonaceous products). The control experiments demonstrate that there is no thermal-catalytic contribution (Figure S6a), indicating that the coupling of CH_4_ is a photocatalytic reaction rather than a photothermal catalytic reaction. Furthermore, the Pd_5_/Zn_0.35_-WO_3_ catalyst can still effectively realize selective photocatalytic nonoxidative coupling of CH_4_ to C_2_H_4_ outside the laboratory under condensed sunlight (Figure S6a), indicating its great potential for practical application. Such performance well exceeds the activity and selectivity of the state-of-the-art catalysts for photocatalytic nonoxidative coupling of CH_4_ to C_2_H_4_ (Table S1). The yields of C_2_H_4_ and C_2_H_6_ for Pd_5_/Zn_0.35_-WO_3_ are 19-fold and 5.5-fold higher than that of bare WO_3_, confirming that the introduced Zn and Pd give a boost to the activation and coupling of CH_4_. More notably, the yields of H_2_ and carbon-containing products almost conform to the stoichiometric ratio during the CH_4_ conversion process, indicating that the hydrogen atoms are derived from CH_4_ with high atom economy (Figure S6b and Table S2).

Upon recognizing the significantly enhanced catalytic performance, the individual roles of Zn dopants and Pd nanoparticles are explored by investigating the catalytic performance for the control samples with only Zn doping and Pd loading, as well as assessing the effects of their loading amounts on the performance. Compared to bare WO_3_, the Zn-doped WO_3_ exhibits remarkably enhanced C_2_ products yield and C_2_H_4_ selectivity, and the C_2_H_4_ yield increases with the amount of doped Zn within a certain range (Figure 2(a)), suggesting that the doped Zn can facilitate CH_4_ activation and coupling. However, excessive Zn doping leads to the decrease in C_2_ products yield, most likely due to the increased work function and reduced lattice oxygen content by excessive doped Zn [31]. The activation of CH_4_ heavily depends on the O^−^ centers in Zn^+^−O^−^ pairs. In the absence of Pd, the O^−^ centers in Zn_0.35_-WO_3_ serving as strong oxidants cause the serious overoxidation of activated ·CH_3_ to CO_2_ (Figure S6b). The Pd nanoparticles-modified WO_3_ (Pd_5_/WO_3_) also exhibits substantially enhanced C_2_ products yield and C_2_H_4_ selectivity, while a considerable amount of C_3_H_6_ emerges. The production of C_3_H_6_ indicates that the introduced Pd nanoparticles serving as active sites have a strong dehydrogenation capability to further dehydrogenate methyl intermediates to generate methylene and methyne radicals, which undergo cross-coupling reaction to produce C_3_H_6_. Furthermore, after modifying Zn_0.35_-WO_3_ with Pd nanoparticles, the production yield of C_2_H_4_ further increases significantly with the loading amount of Pd (Figure 2(a)). This verifies that the modified Pd nanoparticles are conducive to dehydrogenating methyl radicals and further promoting the generated radicals to undergo self- and cross-coupling reactions, which eventually dramatically suppresses the overoxidation of carbon intermediates to CO_2_ as compared with Zn_0.35_-WO_3_ (Figure S6b). Moreover, excessive Pd loading, such as Pd_7_/Zn_0.35_-WO_3_, leads to the reduced yields of C_2_H_6_ and C_2_H_4_ without production of C_3_H_6_, most likely because the agglomeration of small Pd nanoparticles weakens their dehydrogenation effect. The results above demonstrate the significant roles of synergistic Pd and Zn on WO_3_ in promoting the CH_4_ coupling and C_2_H_4_ production as well as suppressing the overoxidation.

To evaluate the stability of the model photocatalyst, the physicochemical properties of Pd_5_/Zn_0.35_-WO_3_ after reaction are investigated. After the reaction, the Pd_5_/Zn_0.35_-WO_3_ sample exhibits slight changes in color, crystallinity, and light absorption properties, but no obvious change on morphology can be observed (Figure S7a-d). Such changes are related to the consumption of a small amount of lattice oxygen in the sample during the reaction, as evidenced by O 1 s XPS spectra (Figure S7e). The production of CO_2_ (Figure S6b) under oxygen-free reaction conditions also proves that the lattice oxygen could be consumed in the process of CH_4_ overoxidation. Nevertheless, such lattice oxygen consumption would not limit the long-term application of photocatalyst; the consumed lattice oxygen can be effectively replenished after photo-oxidation treatment under air conditions by seizing oxygen atoms from the environment. The color, crystallinity, and light absorption properties are almost restored to the state of fresh sample after the treatment (Figure S7). These results suggest that the model photocatalyst can maintain the recyclability through the batch reaction mode. To further assess the recoverability and recyclability of our model photocatalyst, the cycling tests are performed on the Pd_5_/Zn_0.35_-WO_3_ catalyst, during which a photo-oxidation pretreatment of 30 min is performed on the recycled catalyst under air conditions before each cycle (Figure 2(b)). After the six cycles, the recycled catalyst well retains the activity and selectivity for photocatalytic coupling of CH_4_ to C_2_H_4_, manifesting the eminent recyclability and practicability of our model photocatalyst.

To further verify the carbon source of produced C_2_H_4_ and C_2_H_6_ in photocatalytic CH_4_ conversion, isotope labeling experiment is performed by using ^13^CH_4_ as the reactant. The ^13^C_2_H_4_, ^13^C_2_H_6_, and ^3^CO_2_ products as well as various intermediates derived from isotopic ^13^CH_4_ can be observed by gas chromatography-mass spectrometry (GC-MS, Figure 2(c) and S8). Additionally, no products are detected during the control experiments without catalyst or under dark condition (Figure 2(a)). These results confirm that all coupling products are derived from CH_4_, rather than the release of any residual organic matters in raw materials during the preparation process.

**Figure 3:**
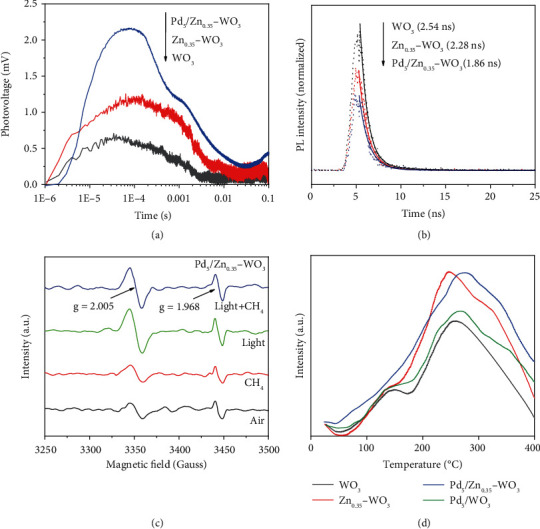
Charge dynamics and CH_4_ adsorption behavior. (a) TR-SPV responses of WO_3_, Zn_0.35_-WO_3_, and Pd_5_/Zn_0.35_-WO_3_. (b) TS-PL spectra of WO_3_, Zn_0.35_-WO_3_, and Pd_5_/Zn_0.35_-WO_3_. (c) In situ EPR signals of Pd_5_/Zn_0.35_-WO_3_ collected under different conditions. (d)) CH_4_-TPD curves of WO_3_, Zn_0.35_-WO_3_, Pd_5_/WO_3_, and Pd_5_/Zn_0.35_-WO_3_.

To elucidate the origin of the superior activity by our designed photocatalyst, we further survey its charge dynamics behavior, which is a key factor to the photocatalytic efficiency. The time-resolved surface photovoltage (TR-SPV), as an advanced characterization method to comprehend the behavior of charge separation and transfer, can qualitatively estimate the charge separation efficiency and photogenerated carrier lifetime by analyzing the signal intensity and duration. To put it simply, the stronger TS-SPV signal, the better charge separation; the wider TS-SPV signal, the longer charge lifetime. For the Pd_5_/Zn_0.35_-WO_3_ with Zn doping and subsequent Pd modification on WO_3_, both the intensity and duration of the TS-SPV signal gradually increase (Figure 3(a)), suggesting the enhanced charge separation efficiency and photogenerated carrier lifetime by introducing Zn and Pd. Such a positive effect on charge dynamics is also confirmed by the steady-state surface photovoltage spectroscopy (Figure S9). Moreover, the transient-state photoluminescence (TS-PL) spectroscopy (Figure 3(b) and Table S3) is further employed to reveal the dynamic charge behavior by fluorescence lifetimes. The fluorescence lifetime for Pd_5_/Zn_0.35_-WO_3_ (*τ* = 1.86 ns) is the shortest among the samples (WO_3_, *τ* = 2.54 ns; Zn_0.35_-WO_3_, *τ* = 2.28 ns). This indicates that the photogenerated charges in Pd_5_/Zn_0.35_-WO_3_ are favorably captured by doped Zn and modified Pd during the charge migration process, thus achieving the promoted charge separation. Overall, the results above confirm the role of Pd-Zn comodification in facilitating charge separation and transfer, which consequently promotes CH_4_ conversion reaction.

Since the promoting effect of Pd-Zn comodification on charge separation and transfer is clarified, in situ electron paramagnetic resonance (EPR) technology is used to understand the specific charge migration process. As shown in the EPR spectra (Figure 3(c) and S10), the intensity of Zn^+^ signal at g = 1.968 for Pd_5_/Zn_0.35_-WO_3_ increases upon light irradiation, manifesting that the photogenerated electrons are transferred from WO_3_ to doped Zn^2+^ to produce Zn^+^ sites [32]. In addition, the signals at g = 2.005 attributed to unpaired electrons trapped in surface defects (V_O_^+^ or O^−^) become stronger for both WO_3_ and Pd_5_/Zn_0.35_-WO_3_ upon light irradiation. Considering that Zn^+^ and O^−^ are always generated in pairs, the enhanced signals at g = 2.005 for Pd_5_/Zn_0.35_-WO_3_ suggest the emergence of photogenerated holes-enriched lattice oxygen sites (O^−^ centers) [22, 28]. Meanwhile, the enhanced signals at g = 2.005 for WO_3_ can be attributed to the unpaired photogenerated electrons trapped in oxygen vacancies, as there is no obvious signal for lattice electron trapping sites. Upon introducing CH_4_, the signal intensity of O^−^ centers at g = 2.005 slightly decreases for Pd_5_/Zn_0.35_-WO_3_ under light irradiation, indicating that the O^−^ centers play a vital role for CH_4_ activation and the free electrons and holes are continually supplied under light irradiation to maintain the content of Zn^+^−O^−^ pairs (which have been well recognized as efficient active sites for CH_4_ activation [21–23]) for achieving efficient catalytic CH_4_ conversion. In contrast, the signal intensity at g = 2.005 does not obviously change for WO_3_ upon introducing CH_4_ under light irradiation, which is consistent with the poor activity for photocatalytic CH_4_ conversion. This reveals that WO_3_ lacks sufficient O^−^ centers to activate CH_4_ without Zn doping. The in situ EPR results confirm the specific electrons/holes migration path from WO_3_ to doped Zn^2+^/lattice O and the emergence of Zn^+^−O^−^ paired sites, which play an important role in adsorbing and activating the CH_4_ molecules.

Upon ascertaining the charge dynamics behavior, we further examine the role of introduced Zn and Pd in CH_4_ activation and coupling process. The CH_4_ temperature programmed desorption (CH_4_-TPD) measurements are first performed to explore the CH_4_ adsorption behavior, which is the essential prerequisite for activation process. As shown by the CH_4_-TPD curves (Figure 3(d)), two wide gas desorption peaks, corresponding to physical adsorption and chemical adsorption, appear, respectively, in the low temperature range (100-150°C) and the middle temperature range (200-400°C) for WO_3_. After doping Zn sites, Zn_0.35_-WO_3_ shows similar physical adsorption peak in low temperature range, while the chemical desorption peak area in middle temperature range increases significantly as compared with WO_3_, indicating that more CH_4_ is firmly adsorbed on the catalyst surface by chemisorption. More importantly, a new CH_4_ chemical desorption peak appears around 325°C after Zn doping, suggesting that the doped Zn sites can promote the CH_4_ adsorption capacity, thereby contributing to the enhanced CH_4_ conversion performance. When the Pd nanoparticles are incorporated into the catalyst, the CH_4_ chemical desorption temperature for Pd_5_/WO_3_ and Pd_5_/Zn_0.35_-WO_3_ shifts toward higher temperature by about 25°C, and the peak area further increases. This indicates that the additional Pd nanoparticles can further enhance the CH_4_ adsorption capacity through increasing the binding strength for CH_4_. Combined with mass spectra (MS), the CH_4_ TPD-MS technique is further used to analyze the related species derived from CH_4_ dissociation. As the essential product in CH_4_ conversion, the peak area for H_2_ is significantly enhanced after Zn doping (Figure S11), especially after Pd-Zn comodification as compared with WO_3_, demonstrating that the modified Pd nanoparticles exhibit a stronger effect on dehydrogenation of the intermediates, contributing to the increased H_2_ production. The results above indicate that the doped Zn plays a major role in promoting the CH_4_ adsorption, while the modified Pd nanoparticles play a dominant role in facilitating the dehydrogenation of the intermediates.

**Figure 4:**
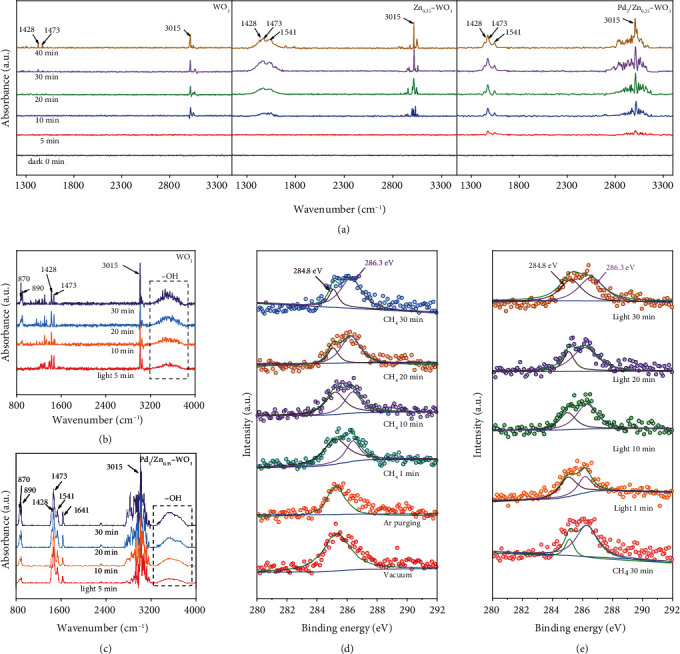
In situ spectroscopic measurements for tracking the evolution of intermediates during photocatalytic CH_4_ conversion. (a) In situ DRIFTS spectra of CH_4_ adsorption in dark for different times over WO_3_, Zn_0.35_-WO_3_, and Pd_5_/Zn_0.35_-WO_3_. (b and c) In situ DRIFTS spectra of CH_4_ conversion under light irradiation for different times over WO_3_ and Pd_5_/Zn_0.35_-WO_3_. (d and e) In situ NAP-XPS spectra of C 1 s over Pd_5_/Zn_0.35_-WO_3_ upon exposure to 0.45 mbar of CH_4_ in dark and under light irradiation for different times.

To further decode the reaction mechanism, in situ diffuse reflectance infrared Fourier-transform spectroscopy (DRIFTS) and in situ near ambient pressure x-ray photoelectron spectroscopy (NAP-XPS) are employed to track the evolution of intermediates during the CH_4_ activation and coupling process. As shown in the in situ DRIFTS spectra for WO_3_, Zn_0.35_-WO_3_, and Pd_5_/Zn_0.35_-WO_3_ (Figures 4(a)–4(c)), when the photocatalysts are fully immersed in the CH_4_ atmosphere for 40 min under dark conditions (Figure 4(a)), the absorption peaks at 1428, 1473, and 3015 cm^−1^, corresponding to the symmetrical and asymmetrical deformation vibration of the C-H bond in CH_4_ molecules, emerge and increase with the adsorption time [21, 27, 33–35]. This implies that the CH_4_ molecules are increasingly adsorbed on the photocatalyst surface. After modification of Zn and Pd, the intensity of each CH_4_-related absorption peak for Zn_0.35_-WO_3_, and Pd_5_/Zn_0.35_-WO_3_ in the same adsorption time period is significantly promoted as compared with WO_3_. This observation is particularly emphasized on the peak for the symmetric vibration of the C-H bond in CH_4_ molecules (1541 cm^−1^), proving that more CH_4_ molecules are captured on photocatalyst surface attributed to the modified Zn and Pd. In addition, when the adsorption time exceeds 30 min, the peak intensity for CH_4_ has no obvious increase, suggesting that a saturated adsorption has been achieved.

Furthermore, the light irradiation is introduced to examine the CH_4_ coupling reaction process (Figures 4(b) and 4(c)). With the increased light irradiation time, the in situ DRIFTS spectra for Pd_5_/Zn_0.35_-WO_3_ show that the intensities of various CH_4_-related peaks have no obvious change, indicating that the adsorption sites can continuously capture CH_4_ from the environment to maintain the adsorption saturation state during the CH_4_ coupling process (Figure 4(c)). In stark contrast, multiple peaks at 870, 890 cm^−1^, and in the range of 3200-3600 cm^−1^ corresponding to the C-C bond and -OH groups of intermediates appear upon light irradiation, and their intensities gradually increase with the light irradiation time, indicating that the adsorbed CH_4_ has undergone a cleavage and coupling process to generate C_2_H_6_ [36]. Remarkably, the peak at 1641 cm^−1^ in the in situ DRIFTS spectra for Pd_5_/Zn_0.35_-WO_3_, assigned to C=C of methylene radicals, suggests the process that the adsorbed CH_4_ is dehydrogenated, and coupled to produce C_2_H_4_ [37].

In situ NAP-XPS studies are further performed to supplement the information for the evolution of intermediates during the CH_4_ coupling reaction. The corresponding high-resolution C 1 s XPS spectrum of Pd_5_/Zn_0.35_-WO_3_ displays a peak at 284.8 eV under vacuum condition (Figure 4(d)), due to the exogenous residual carbon on photocatalyst surface [21, 37]. Although such residual carbon cannot be completely purged by the Ar flow, the peak intensity basically maintains unchanged and thus has no interference to the subsequent measurement. With the continuous CH_4_ adsorption and accumulation, the intensity of the peak at 286.3 eV assigned to methoxy intermediates increases significantly, indicating that the generated methyl intermediates are adsorbed on lattice oxygen sites. The peak intensity for the methoxy intermediates significantly decreases upon light irradiation (Figure 4(e)), suggesting that the methoxy intermediates are consumed by participating in the following reaction. With the continuous light irradiation, the peak intensities for the methoxy intermediates are recovered, indicating that the methoxy intermediates can be continuously supplemented for maintaining the CH_4_ coupling reaction [38–40].

**Figure 5:**
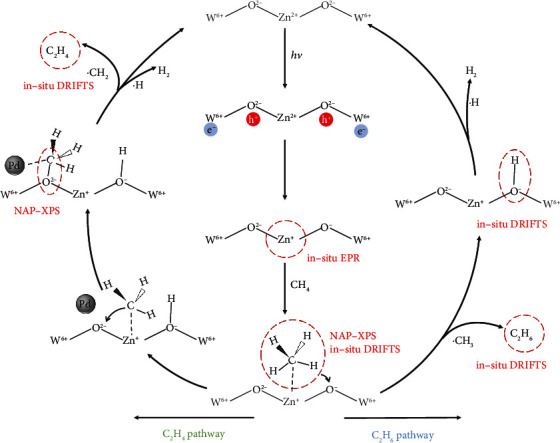
Schematic diagram illustrating the pathway for photocatalytic coupling conversion of CH_4_ to C_2_H_4_ and C_2_H_6_ on the designed Pd_5_/Zn_0.35_-WO_3_ catalyst.

Based on the information gleaned above, the roles of modified Zn and Pd on charge dynamics and reaction intermediates evolution have been elucidated. In addition, the photogenerated holes in the valence band maximum of WO_3_ are thermodynamically feasible for driving the oxidation of CH_4_ to methyl radical (Figure S12) [41]. As such, a reasonable reaction pathway including the photogenerated charge transfer path can be proposed as illustrated in Figure 5. Upon light irradiation, the photogenerated holes are enriched at lattice oxygen (O^2-^) sites to form O^−^ centers, while the photogenerated electrons are transferred to the nearby doped Zn^2+^ sites via W^6+^, forming Zn^+^−O^−^ pairs. The formed Zn^+^ sites are available for capturing the CH_4_ molecules through donating the single electron to the empty C−H *σ*^∗^-antibonding orbital of CH_4_ molecule, while the O^−^ centers have a strongly attractive force to abstract the H atoms from CH_4_ [22, 23]. Subsequently, the adsorbed CH_4_ is activated by Zn^+^−O^−^ pairs to generate methyl intermediates, which are then stabilized by the Zn^+^ sites. The formed methyl intermediates can either follow the C_2_H_6_ pathway after desorbing from the catalyst to generate methyl radicals, or undergo the C_2_H_4_ pathway by diffusing onto the adjacent O^2-^ sites and being further dehydrogenated by Pd nanoparticles to generate methylene radicals. Finally, the produced free methyl radicals and methylene radicals undergo self-coupling reactions to produce the products of C_2_H_4_ and C_2_H_6_. Simultaneously, the H atoms dissociated from the activated CH_4_ can couple with each other to produce H_2_. The holes enriched at O^−^ centers can recombine with the electrons trapped at Zn^+^ sites to generate O^2-^ and Zn^2+^. It should be noted that the lattice oxygen participates in the photocatalytic CH_4_ conversion in two different pathways. One is producing O^−^ centers as active sites to activate CH_4_ molecules for photocatalytic nonoxidative coupling of CH_4_ with a stoichiometric H_2_ evolution, during which the lattice oxygen will not be consumed. The other is serving as strong oxidants leading to the over oxidation of CH_4_ to CO_2_, during which the lattice oxygen will be consumed similarly to the Mars-van Krevelen mechanism [42]. Nevertheless, the consumed lattice oxygen during the overoxidation process can be effectively supplemented by the photo-oxidation treatment under air conditions, realizing the recycling of the model Pd_5_/Zn_0.35_-WO_3_ photocatalyst.

## 3. Discussion

In summary, we have designed Pd-Zn comodified WO_3_ nanosheets as advanced photocatalysts for efficient CH_4_ nonoxidative coupling to C_2_H_4_ with high selectivity under ambient conditions and moderate light irradiation. The optimized Pd_5_/Zn_0.35_-WO_3_ nanostructure, featuring the Zn and Pd as synergistic active sites, achieves superior catalytic performance as compared to the state-of-the-art catalysts for photocatalytic nonoxidative coupling of CH_4_ to C_2_H_4_. Importantly, a nearly stoichiometric yield of valuable H_2_ also has been obtained, suggesting the giant economic incentives. Based on structural characterization and in situ spectroscopic analysis, the remarkable performance of our model photocatalyst is ascribed to the synergized adsorption, activation, and dehydrogenation of CH_4_ by the synergistic O^−^, Zn^+^, and Pd^0^ sites. Specifically, the doped Zn sites play a major role in promoting the adsorption and activation of CH_4_ molecules, while the Pd sites play a dominant role in facilitating the dehydrogenation of the intermediates and suppressing overoxidation. The synergistic functions dramatically increase the selectivity toward C_2_H_4_ and efficiently suppress the CH_4_ overoxidation to CO_2_. This work provides insights for designing highly efficient photocatalyst for selective photocatalytic nonoxidative coupling of CH_4_ toward the high value-added hydrocarbon products through leveraging synergistic catalytic sites to synergize the adsorption, activation, and dehydrogenation processes.

## 4. Materials and Methods

### 4.1. Synthesis of WO_3_ Nanosheets

0.222 g of polyethylene oxide-polypropylene oxide-polyethylen (P123) (Pluronic, M = 5800) was dissolved in 14.444 g of absolute ethanol, and this mixture was stirred continuously for 1 h. After that, deionized water was added into the mixture and stirred for another 1 h. Subsequently, 1.5 mL of ethylene glycol was added to the above mixture and stirred for another 2 h to form a clear solution. The obtained solution was sealed and kept in a brown reagent bottle at least 48 h before further use (denoted as solution A). Subsequently, 0.444 g of WCl_6_ was added into 16.833 g solution A, and stirred for 20 min to obtain a yellow solution. The solution was then transferred to a 50 mL teflon-lined stainless autoclave. The sealed autoclave was kept under 110°C for 3 h and naturally cooled to room temperature. After the solvothermal treatment, the blue precipitate was washed with absolute ethanol and dried in a vacuum oven at 80°C for 12 h. Then, the precipitate was ground to obtain powder. Finally, the powder was placed in a muffle furnace and calcinated at 400°C for 1 h to obtain the WO_3_ nanosheets with a yellow color.

### 4.2. Synthesis of Zn-Doped WO_3_ Nanosheets

The Zn-doped WO_3_ nanosheets were synthesized by following the same procedure for WO_3_ nanosheets except that various mass (0.005, 0.010, and 0.020 g) of Zn (Ac)_2_ were added into the solution A with WCl_6_ at the same time. The prepared Zn-doped WO_3_ nanosheets with various mass fraction of Zn are denoted as Zn_X_-WO_3_, where *X* refers to the theoretical mass fraction of Zn in the sample.

### 4.3. Synthesis of Pd-Zn Comodified WO_3_ Nanosheets

1.000 g optimized Zn_0.35_-WO_3_ and different amount (0.063, 0.105, and 0.147 g) of Pd (Ac)_2_ were dispersed in 50 mL of CH_2_Cl_2_ under stirring for 24 h at room temperature [43]. After separation, washing, and drying, the powder of Pd-Zn comodified WO_3_ nanosheets was obtained and denoted as Pd_Y_/Zn_0.35_-WO_3_, where *Y* refers to the mass fraction of Pd in the sample.

### 4.4. Evaluation of Photocatalytic Performance for CH_4_ Conversion

In the photocatalytic CH_4_ conversion test, the dispersion (2 mg photocatalyst, and 500 *μ*L H_2_O) was evenly daubed on a FTO conductive glass (1 cm × 4 cm). After the photocatalyst was dried, the FTO conductive glass was put into a 67 mL quartz reaction tube filled with CH_4_ (99.999%). Subsequently, the reactor was irradiated vertically with a 300 W xenon lamp for 2 h with a light intensity of 500 mW/cm^2^ and a irradiation area of 2 cm^2^. A gas chromatography (GC) of GC-7890B (Agilent) with TCD and flame ionization detector (FID) was used to detect H_2_ and other hydrocarbon products (C_2_H_6_ and C_2_H_4_). Other overoxidation products (CO and CO_2_) were analyzed by GC-7890A gas chromatography (Agilent) with TCD and FID detectors. Using the same method, isotope labelling experiments were performed with ^13^CH_4_ as the reactant and gas chromatography-mass spectrometry (GC-MS, 7890A-5975C, Agilent) as the detection instrument.

## Data Availability

All the data needed to evaluate the conclusions in the paper are present in the paper and in the Supplementary Materials. Additional data related to this paper may be requested from the authors.

## Supplementary Materials

Supplementary Materials Figure S1. (a) XRD patterns and (b) UV-vis DRS spectra of WO_3_, Zn_0.35_-WO_3_, and Pd_5_/Zn_0.35_-WO_3_ samples. Figure S2. SEM images of (a, b) WO_3_, (c) Zn_0.35_-WO_3_, and (d) Pd_5_/Zn_0.35_-WO_3_. Figure S3. (a) W 4f and (b) O 1 s XPS spectra of WO_3_, Zn_0.35_-WO_3_, and Pd_5_/Zn_0.35_-WO_3_. (c) Zn 2p XPS spectra of Zn_0.35_-WO_3_ and Pd_5_/Zn_0.35_-WO_3_. (d) Pd 3d XPS spectra of Pd_5_/Zn_0.35_-WO_3_. Figure S4. GC data for photocatalytic CH_4_ conversion over (a) WO_3_, (b) Zn_0.35_-WO_3_, and (c) Pd_5_/Zn_0.35_-WO_3_. Figure S5. Time-dependent production yield of C_2_H_4_, C_2_H_6_, and C_3_H_6_ in photocatalytic CH_4_ conversion over Pd_5_/Zn_0.35_-WO_3_ photocatalysts under light irradiation of 2 h. Figure S6. Products yields and theoretical H_2_ yield for CH_4_ conversion over WO_3_, Zn_0.35_-WO_3_, and Pd_5_/Zn_0.35_-WO_3_ photocatalysts under light irradiation of 2 h. Figure S7. (a) Photographs of the Pd_5_/Zn_0.35_-WO_3_ sample before reaction, after reaction and after photo-oxidation treatment. (b) SEM image of Pd_5_/Zn_0.35_-WO_3_ after photo-oxidation treatment. (c) XRD patterns, (d) UV-vis DRS spectra, and (e) O 1 s XPS spectra of Pd_5_/Zn_0.35_-WO_3_ before reaction, after reaction, and after photo-oxidation treatment. Figure S8. GC-MS data of ^13^CO_2_ produced in photocatalytic ^13^CH_4_ coupling by Pd_5_/Zn_0.35_-WO_3_. Figure S9. SS-SPS responses of WO_3_, Zn_0.35_-WO_3_, and Pd_5_/Zn_0.35_-WO_3_. Figure S10. In situ EPR signals of WO_3_ collected under different conditions. Figure S11. TPD-MS profiles of H_2_ over WO_3_, Zn_0.35_-WO_3_, and Pd_5_/Zn_0.35_-WO_3_. Figure S12. Illustration of the band structures of WO_3_ and the redox potentials for oxidizing CH_4_ to·CH_3_. Table S1. The comparison of catalytic performance with representative state-of-the-art photocatalysts for photocatalytic coupling of CH_4_ to C_2_ compounds. Table S2. The yields of products in photocatalytic CH_4_ conversion over Pd_5_/Zn_0.35_-WO_3_. Table S3. Fluorescence lifetimes related to the TS-PL spectra of WO_3_, Zn_0.35_-WO_3_, and Pd_5_/Zn_0.35_-WO_3_.
