# Supplementary material for: Highly Efficient and Selective Photocatalytic Nonoxidative Coupling of Methane to Ethylene over Pd-Zn Synergistic Catalytic Sites
Source: Research (Wash D C). 2022 Nov 7;2022:9831340. doi: 10.34133/2022/9831340 (PMC9680520; doi:10.34133/2022/9831340)
Supplement: Supplementary Materials — Figure S1. (a) XRD patterns and (b) UV-vis DRS spectra of WO3, Zn0.35-WO3, and Pd5/Zn0.35-WO3 samples. Figure S2. SEM images of (a, b) WO3, (c) Zn0.35-WO3, and (d) Pd5/Zn0.35-WO3. Figure S3. (a) W 4f and (b) O 1 s XPS spectra of WO3, Zn0.35-WO3, and Pd5/Zn0.35-WO3. (c) Zn 2p XPS spectra of Zn0.35-WO3 and Pd5/Zn0.35-WO3. (d) Pd 3d XPS spectra of Pd5/Zn0.35-WO3. Figure S4. GC data for photocatalytic CH4 conversion over (a) WO3, (b) Zn0.35-WO3, and (c) Pd5/Zn0.35-WO3. Figure S5. Time-dependent production yield of C2H4, C2H6, and C3H6 in photocatalytic CH4 conversion over Pd5/Zn0.35-WO3 photocatalysts under light irradiation of 2 h. Figure S6. Products yields and theoretical H2 yield for CH4 conversion over WO3, Zn0.35-WO3, and Pd5/Zn0.35-WO3 photocatalysts under light irradiation of 2 h. Figure S7. (a) Photographs of the Pd5/Zn0.35-WO3 sample before reaction, after reaction and after photo-oxidation treatment. (b) SEM image of Pd5/Zn0.35-WO3 after photo-oxidation treatment. (c) XRD patterns, (d) UV-vis DRS spectra, and (e) O 1 s XPS spectra of Pd5/Zn0.35-WO3 before reaction, after reaction, and after photo-oxidation treatment. Figure S8. GC-MS data of 13CO2 produced in photocatalytic 13CH4 coupling by Pd5/Zn0.35-WO3. Figure S9. SS-SPS responses of WO3, Zn0.35-WO3, and Pd5/Zn0.35-WO3. Figure S10. In situ EPR signals of WO3 collected under different conditions. Figure S11. TPD-MS profiles of H2 over WO3, Zn0.35-WO3, and Pd5/Zn0.35-WO3. Figure S12. Illustration of the band structures of WO3 and the redox potentials for oxidizing CH4 to·CH3. Table S1. The comparison of catalytic performance with representative state-of-the-art photocatalysts for photocatalytic coupling of CH4 to C2 compounds. Table S2. The yields of products in photocatalytic CH4 conversion over Pd5/Zn0.35-WO3. Table S3. Fluorescence lifetimes related to the TS-PL spectra of WO3, Zn0.35-WO3, and Pd5/Zn0.35-WO3. [file 9831340.f1.zip › SI-clean.pdf]

Research
A SCIENCE PARTNER JOURNAL
SPJ
SCIENCE PARTNER JOURNALS

Supporting Information

Title

Highly Efficient and Selective Photocatalytic Nonoxidative Coupling of Methane to Ethylene over Pd-Zn Synergistic Catalytic Sites

Authors

Yanduo Liu[1, 2], Yihong Chen[1], Wenbin Jiang[1], Tingting Kong[3]*, Pedro H. C. Camargo[4], Chao Gao[1]*, and Yujie Xiong[1, 2, 3]*

Affiliations

[1]School of Chemistry and Materials Science, University of Science and Technology of China, Hefei, Anhui 230026, China

[2]Institute of Energy Hefei Comprehensive National Science Center, Hefei, Anhui 230031, China

[3]Key Laboratory of Functional Molecular Solids, Ministry of Education, Anhui Engineering Research Center of Carbon Neutrality, College of Chemistry and Materials Science, Anhui Normal University, Wuhu, Anhui 241000, China

[4]Department of Chemistry, University of Helsinki, FIN-00014, Finland

Correspondence should be addressed to Tingting Kong; 793255775@qq.com; Chao Gao; gaoc@ustc.edu.cn; and Yujie Xiong; yjxiong@ustc.edu.cn

Chemicals

Tungsten chloride (WCl_6, Aladdin, T106507, 98%), Pluronic P123 (Aladdin, P822487, Mn = 5800, 98%), zinc acetate ($Zn(Ac)_2$, Aladdin, Z110780, 99.995%), dichloromethane (CH_2Cl_2, Aladdin, D116149, 99.5%), and palladium acetate ($Pd(Ac)_2$, Aladdin, P111485, 99.9%) were used in photocatalyst preparation. Deuterated methane (CD_4, 99 at.%) was bought from Sigma-Aldrich. All other chemicals were of analytical grade and purchased from Sinopharm Chemical Reagent Co., Ltd. The water used in all experiments was deionized with the resistivity of 18.2 MΩ·cm. All chemicals were used as received without further purification.

Supplementary Methods

X-ray diffraction (XRD)

Powder XRD patterns were recorded by using a Philips Pert Pro Super X-ray diffractometer with Cu-Kα radiation (λ = 1.54178 Å).

UV-vis diffuse reflectance spectroscopy (DRS)

The UV–vis DRS curves were collected using a Model Shimadzu UV-2750 spectrophotometer.

Transmission electron microscopy (TEM)

TEM characterizations were carried out on a JEOL JEM-2010EX instrument, operating at a 200 kV accelerating voltage.

Scanning electron microscopy (SEM)

SEM images were taken using a HitachiS-4800 instrument (Tokyo, Japan), operating at acceleration voltage of 15 kV.

X-ray photoelectron spectroscopy (XPS)

XPS spectra were collected by ESCALAB 250Xi (Thermo Fisher).

Near ambient pressure (NAP)-XPS

NAP-XPS spectra were collected at the SPECS NAP-XPS. The light irradiation was introduced into the analysis chamber through an observation window using a 300 W xenon lamp. All the XPS spectra were calibrated according to the C 1s peak at 284.8 eV.

Steady-state surface photovoltage spectroscopy (SS-SPS)

SS-SPS measurements were carried out with a self-built experimental equipment under the control of different gas atmospheres, which was equipped with a light chopper (SR540, USA) and a double prism monochromator (SBP300).

Time-resolved surface photovoltage (TR-SPV)

TR-SPV measurements were performed with a self-built experimental equipment, equipped with a second harmonic Nd: YAG laser (Lab-130-10H, Newport, Co.), high energy pyroelectric sensor (PE50BF-DIF-C, Ophir Photonics Group) and 1GHz digital phosphor oscilloscope (DPO 4104B, Tektronix).

Transient-state photoluminescence (TS-PL) spectroscopy

TS-PL spectra were measured at a single photon counting spectrometer (Edinburgh Instrument, FLS 920) with 1 μs pulse lamp as the excitation. The time-resolved transient PL decay curve was mathematically described as the following biexponential function:

$$\mathrm{I(t)} = A_1 \exp(-t/\tau_1) + A_2 \exp(-t/\tau_2)$$

where τ_1 and τ_2 are fluorescence lifetimes, and A_1 and A_2 are corresponding amplitudes. The calculation formula for average fluorescent lifetime is described as follows:

$$\tau_a = \frac{A_1\tau_1^2 + A_2\tau_2^2}{A_1\tau_1 + A_2\tau_2}$$

Electron paramagnetic resonance (EPR)

The EPR tests of free radicals were carried out on the Bruker EMX plus model spectrometer.

Temperature programmed desorption (TPD)

TPD measurements were carried out in two patterns. One was to detect the desorption capacity of adsorbed gas (CH_4) on the sample surface on a Tianjin XQ TP5080 auto-adsorption apparatus with a thermal conductivity detector (TCD). During the measurements, 0.050 g sample was first treated at 350 °C for 30 min to remove impurities under He gas flow. After cooling to room temperature, the adsorption gas with a flow rate of 50 mL min^{-1} was introduced for 30 min until it reached the adsorption saturation. After purging with He for 30 min to remove excess gas, the detection curve was recorded when the temperature was raised to 800 °C at a rate of 10 °C min^{-1}.

In situ diffuse reflectance infrared Fourier transform spectroscopy (DRIFTS)

In situ DRIFTS was performed on a Nicolet Is50 spectrometer. Before the measurement, the surface impurities of each sample in the in-situ cell (Harrick) were removed at 175 °C for 30 min in continuous N_2 atmosphere (30 mL min^{-1}, 99.999%). After the treated sample was cooled to room temperature, the background spectrum in the range of 650 to 4000 cm^{-1} was recorded by the MCT/A detector (4 cm^{-1} resolution). In the formal testing phase, CH_4 (30 mL min^{-1}, 99.999%) was used instead of N_2 to continuously introduce into the in-situ cell for 40 min in dark until

the CH_4 adsorption on the photocatalyst surface was saturated. Subsequently, the CH_4 flowing was turned off, and the in-situ cell was irradiated by a 300 W Xe lamp for 30 min. The spectra were recorded at different times in dark and under the light irradiation, and the in-situ DRIFTS spectra were obtained by subtracting the background spectrum.

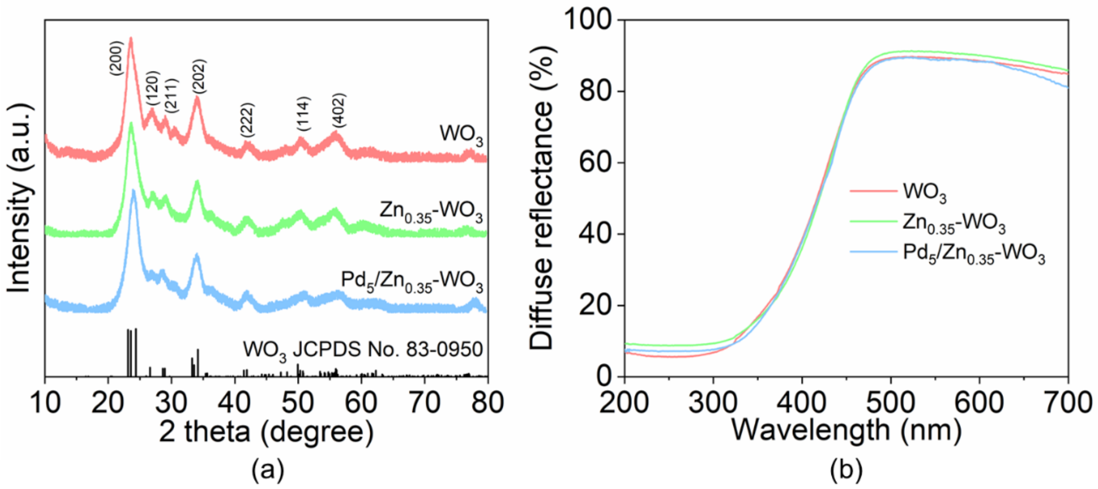

Figure S1. (a) XRD patterns and (b) UV-vis DRS spectra of WO_3, $Zn_{0.35}$-WO_3 and Pd_5/$Zn_{0.35}$-WO_3 samples.

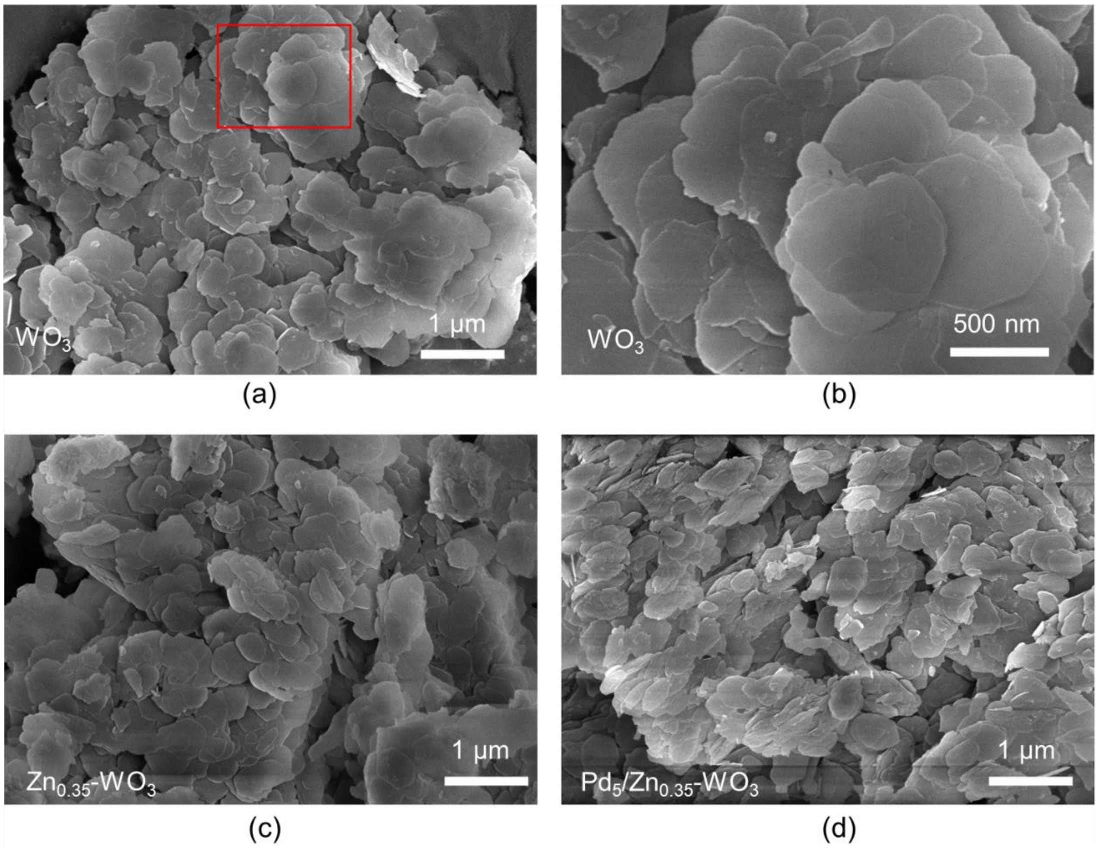

Figure S2. SEM images of (a, b) WO_3, (c) $Zn_{0.35}$-WO_3 and (d) $Pd_5/Zn_{0.35}$-WO_3.

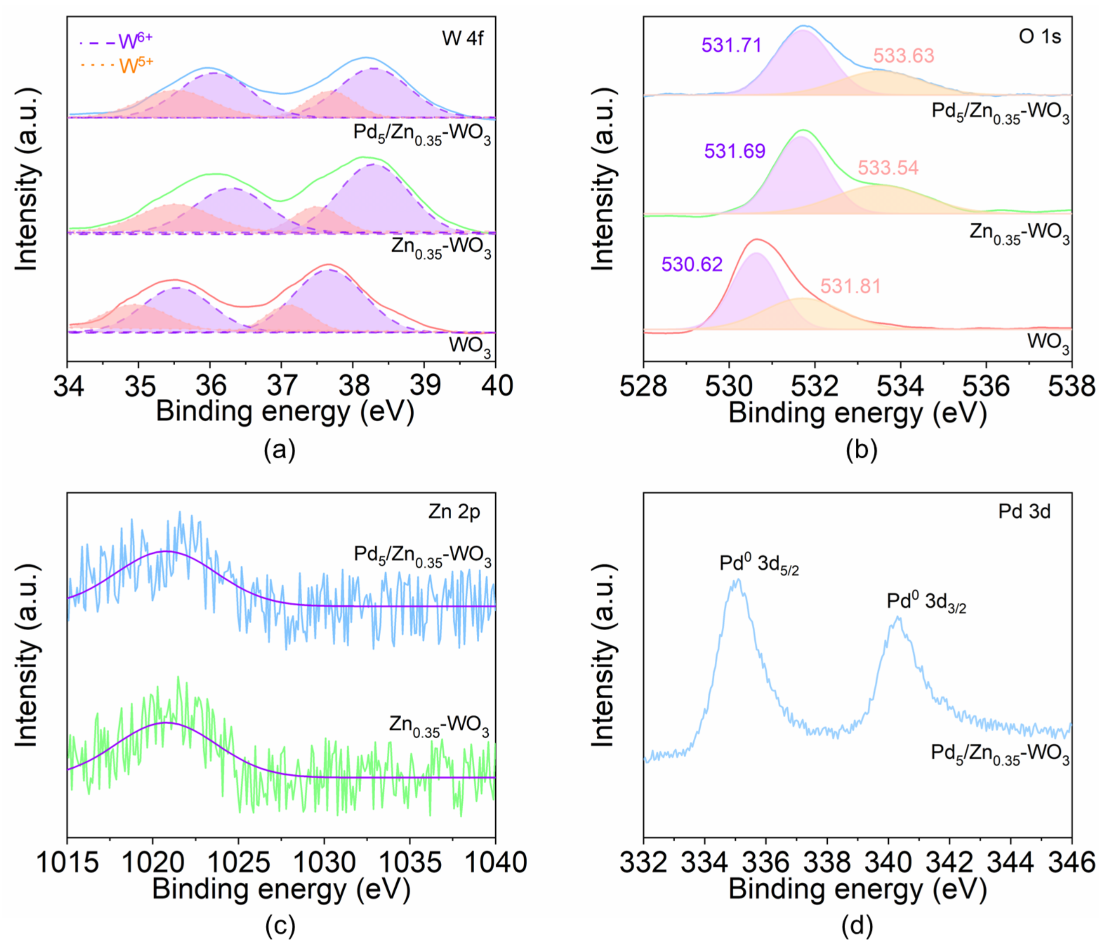

Figure S3. (a) W 4f and (b) O 1s XPS spectra of WO_3, $Zn_{0.35}$-WO_3 and $Pd_5/Zn_{0.35}$-WO_3. (c) Zn 2p XPS spectra of $Zn_{0.35}$-WO_3 and $Pd_5/Zn_{0.35}$-WO_3. (d) Pd 3d XPS spectra of $Pd_5/Zn_{0.35}$-WO_3.

Obvious peaks for low-valence W^{5+} are observed in the W 4f XPS spectra of bare WO_3 (Figure S3a). In the meantime, a peak at the binding energy of 531.8 eV representing the oxygen vacancies is resolved for O 1s XPS spectra (Figure S3b). This indicates that oxygen vacancies exist in WO_3 as defects [S1, S2]. For $Zn_{0.35}$-WO_3 and $Pd_5/Zn_{0.35}$-WO_3, the peaks for W 4f and O 1s shift toward higher binding energies as compared with bare WO_3 (Figure S3a and S3b), while the peaks for Zn 2p shift toward lower binding energies as compared with bare ZnO (Figure S3c). This reflects that isolated Zn^{2+} ions have been successfully doped into the WO_3 lattice and has formed effective chemical bonds with W and O. At the same time, the binding energy of Pd 3d for $Pd_5/Zn_{0.35}$-WO_3 is consistent with that for Pd^0 (Figure S3d), proving that the Pd nanoparticles are indeed formed by the self-reduction method.

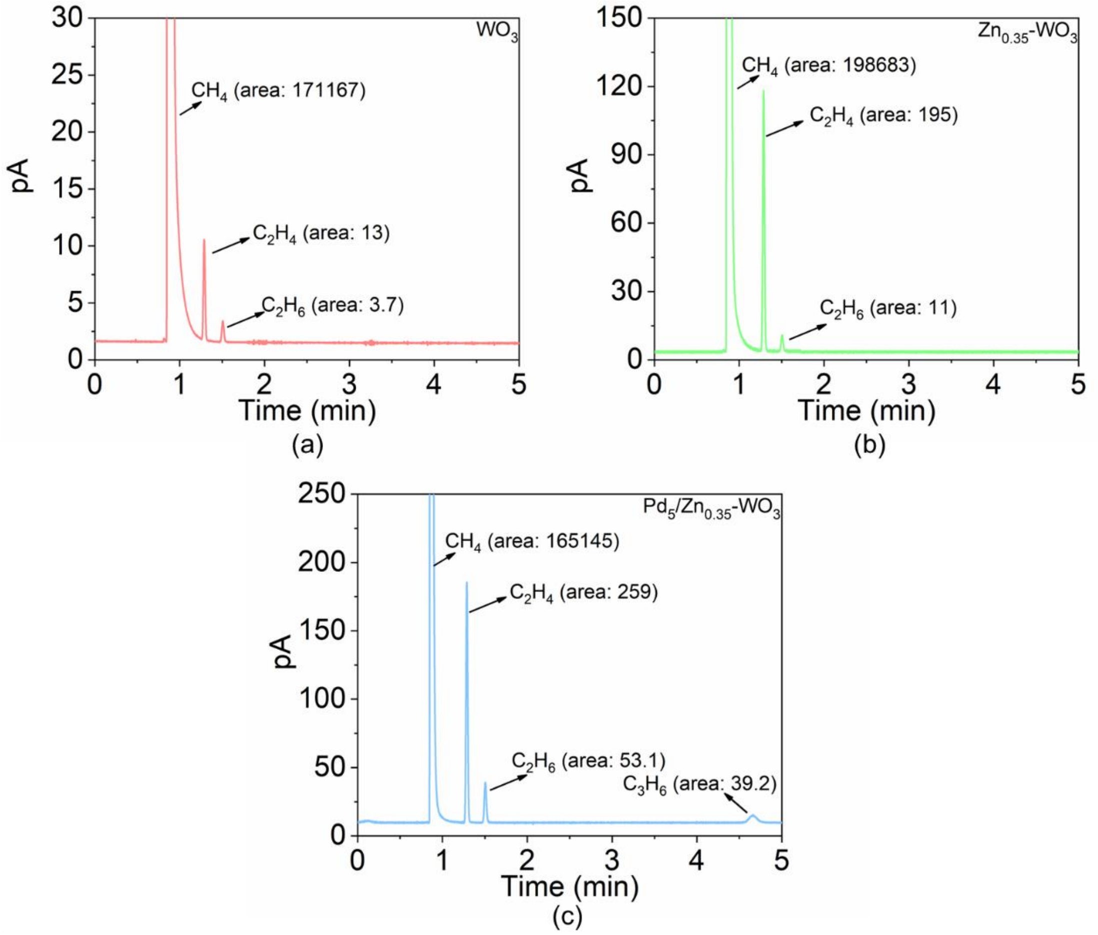

Figure S4. GC data for photocatalytic CH_4 conversion over (a) WO_3, (b) $Zn_{0.35}$-WO_3 and (c) $Pd_5/Zn_{0.35}$-WO_3.

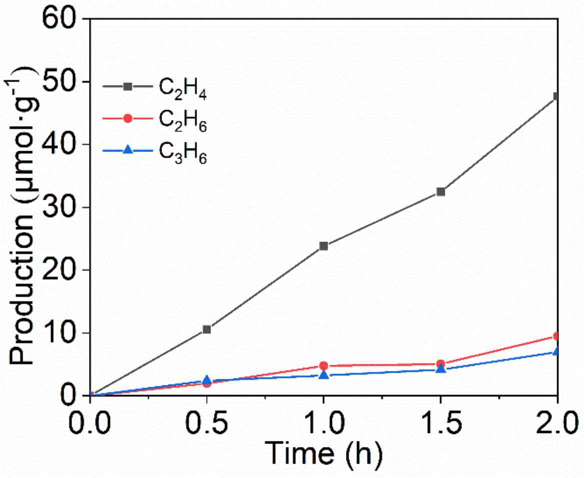

Figure S5. Time-dependent production yield of C_2H_4, C_2H_6 and C_3H_6 in photocatalytic CH_4 conversion over $Pd_5/Zn_{0.35}$-WO_3 photocatalysts under light irradiation of 2 h.

Table S1. The comparison of catalytic performance with representative state-of-the-art photocatalysts for photocatalytic coupling of CH_4 to C_2 compounds.

Catalysts	Production rate of C_2H_4	Production rate of C_2H_6	References
$Pd_5/Zn_{0.35}$-WO_3	24 μmol g^{-1} h^{-1}	5μmol g^{-1} h^{-1}	This work
Au/ZnO	___	11μmol g^{-1} h^{-1}	Energy Environ. Sci. 2018[S3]
Pt/Ga-TiO_2-SiO_2	___	1.57 μmol g^{-1} h^{-1}	JACS 2019[S4]
2Nb-TS	___	1.6μmol g^{-1} h^{-1}	Angew 2021[S5]
ZnO-$AuPd_{2.7\%}$	13.3μmol g^{-1} h^{-1}	21.6 μmol g^{-1} h^{-1}	JACS 2021[S6]
Zn-HPW/TiO_2	___	___	Nat. Commun. 2019[S7]
Ag-HPW/TiO_2	___	20.7μmol g^{-1} h^{-1}	Nat. Energy. 2020[S8]
$Cu_{0.1}Pt_{0.5}$/PC-50	4μmol g^{-1} h^{-1}	64 μmol g^{-1} h^{-1}	JACS 2020[S9]
0.5%Pt/TiO_2	___	55.5 μmol g^{-1} h^{-1}	Appl. Catal. B 2017[S10]
Pt/Ga_2O_3	___	70 μmol g^{-1} h^{-1}	ACS Catal. 2021[S11]
Pd-Bi/Ga_2O_3	___	1.2 μmol g^{-1} h^{-1}	ACS Catal. 2021[S12]

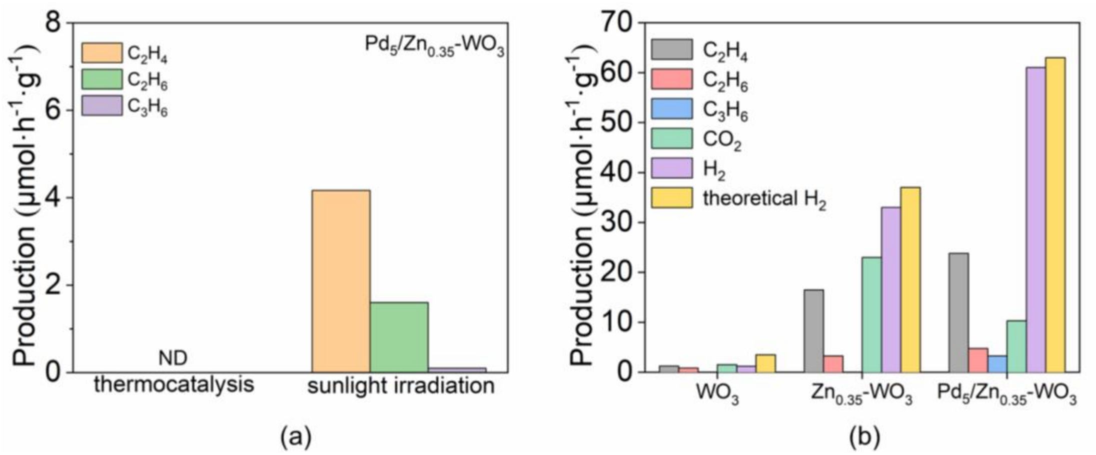

Figure S6. (a) C_{2+} compound yields for CH_4 conversion by $Pd_5/Zn_{0.35}$-WO_3 photocatalyst under a heating temperature of 77 ℃ without light irradiation, and outside the laboratory under condensed sunlight irradiation of 2 h. "ND" stands for "not detected." (b) Product yields and theoretical H_2 yield for CH_4 conversion over WO_3, $Zn_{0.35}$-WO_3 and $Pd_5/Zn_{0.35}$-WO_3 photocatalysts under light irradiation of 2 h.

Under the applied light intensity of 500 mW/cm^2, the actual temperature of the catalyst surface after 2 h illumination is 77 ℃. To clarify whether there is a thermal catalytic contribution, we have performed the methane coupling reaction at the same temperature without light irradiation. The results show that there is no thermal-catalytic contribution at this low temperature (Figure S6a), indicating that the coupling of methane in this work is a photocatalytic reaction rather than a photothermal catalytic reaction.

To examine the catalytic performance of the $Pd_5/Zn_{0.35}$-WO_3 catalyst directly driven by solar energy, we have performed the photocatalytic CH_4 conversion reaction outside the laboratory under condensed sunlight by a Fresnel condenser lens (Conditions: ambient temperature: 29 ℃, photocatalyst surface temperature: 54 ℃, light intensity: 660 mW/cm^2). The $Pd_5/Zn_{0.35}$-WO_3 catalyst can still effectively realize selective photocatalytic nonoxidative coupling of CH_4 to C_2H_4 (Figure S6a), indicating its great potential for practical application. It should be noted that the reduced C_{2+} compound yields under sunlight is due to the smaller irradiation area by using condenser lens compared to that in laboratory experiment (0.38 cm^2 *vs.* 2.0 cm^2).

Table S2. The yields of products in photocatalytic CH_4 conversion over $Pd_5/Zn_{0.35}$-WO_3.

Product	C_2H_4	C_2H_6	C_3H_6	CO_2	H_2
Experimental yield (μmol/g/h)	24	5	3.3	10.3	60.1
Theoretical stoichiometric H_2 yield (μmol/g/h)	48	5	9.8	0	Total: 62.8

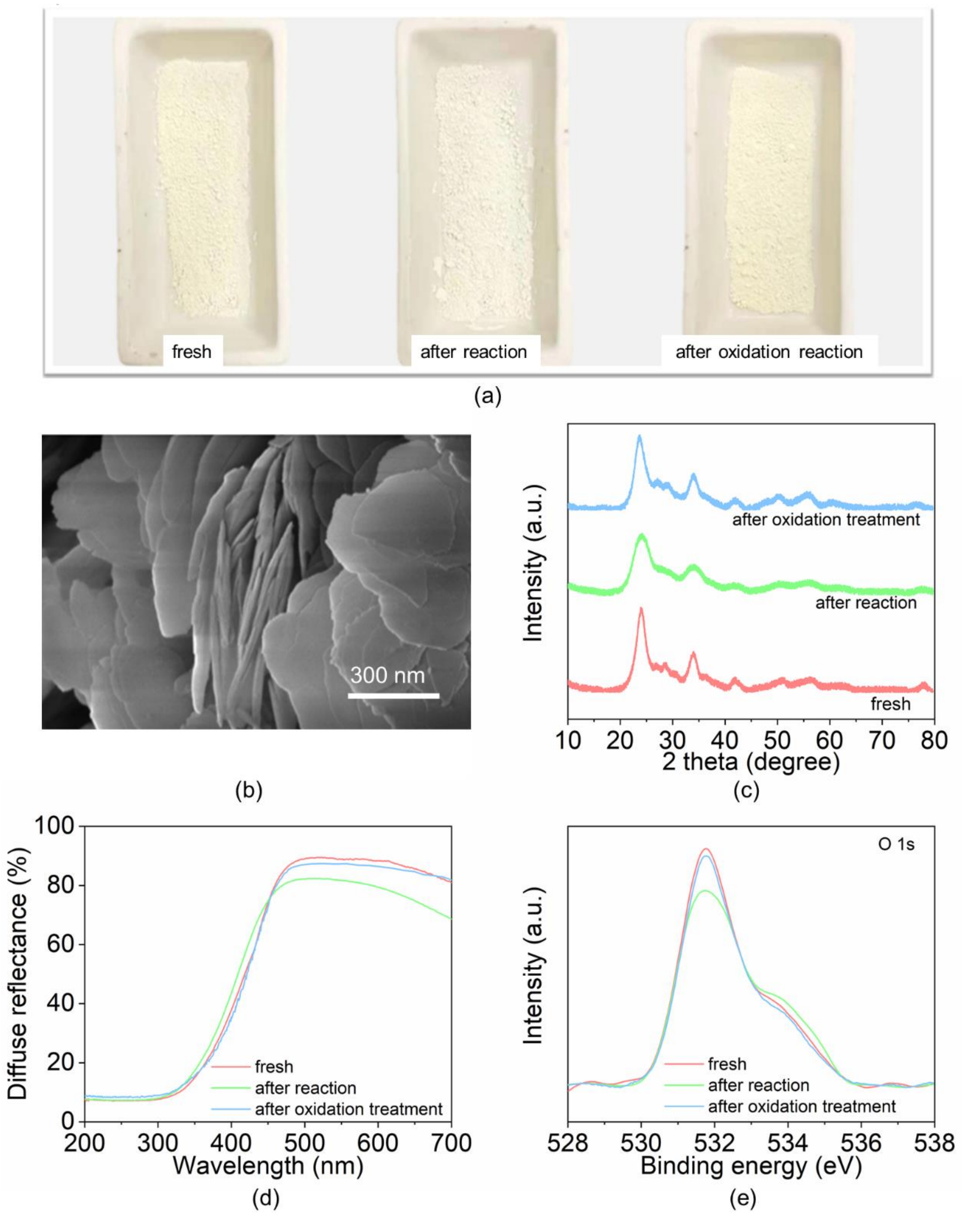

Figure S7. (a) Photographs of the $Pd_5/Zn_{0.35}$-WO_3 sample before reaction, after reaction and after photooxidation treatment. (b) SEM image of $Pd_5/Zn_{0.35}$-WO_3 after photooxidation treatment. (c) XRD patterns, (d) UV-vis DRS spectra and (e) O 1s XPS spectra of $Pd_5/Zn_{0.35}$-WO_3 before reaction, after reaction and after photooxidation treatment.

After the reaction, the color of the $Pd_5/Zn_{0.35}$-WO_3 sample becomes gray as compared with the fresh sample (Figure S7a), and it is recovered to the state of fresh sample after photooxidation treatment under air conditions. SEM image

shows that there is no obvious change on morphology for $Pd_5/Zn_{0.35}$-WO_3 after photooxidation treatment (Figure S7b). Similarly, the $Pd_5/Zn_{0.35}$-WO_3 sample exhibits slight decrease in crystallinity and light absorption properties after reaction (Figure S7c and S7d). However, such changes are almost restored to the state of fresh sample after photooxidation treatment under air conditions. O 1s XPS spectra (Figure S7e) show that the lattice oxygen content decreases after reaction and is almost recovered after photooxidation treatment under air conditions, which is in consistence with the results of XRD and UV-vis DRS characterizations.

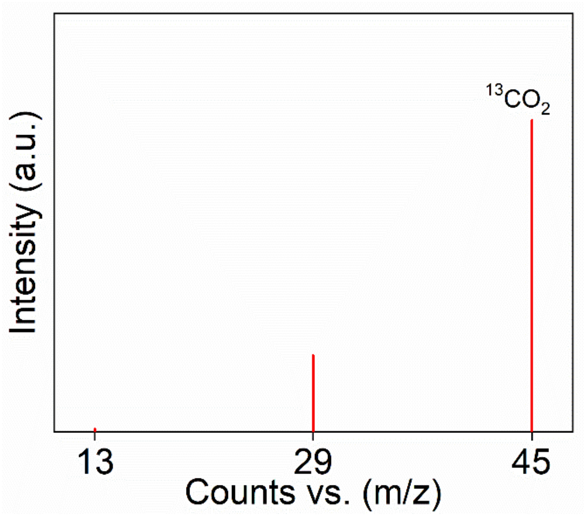

Figure S8. GC-MS data of $^{13}CO_2$ produced in photocatalytic $^{13}CH_4$ coupling by $Pd_5/Zn_{0.35}$-WO_3.

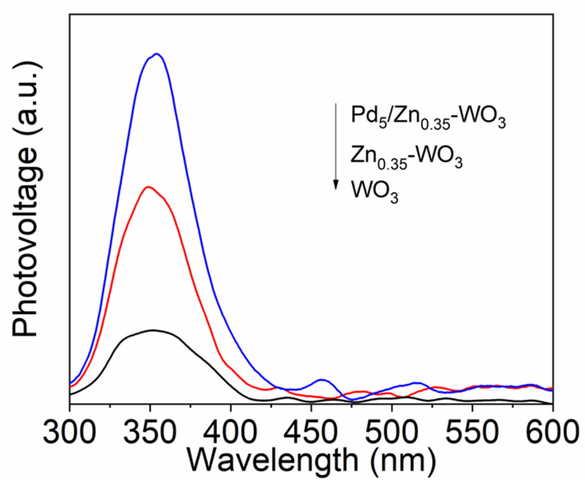

Figure S9. The SS-SPS responses of WO_3, $Zn_{0.35}$-WO_3 and Pd_5/$Zn_{0.35}$-WO_3.

The SS-SPS spectra show that the photovoltage intensities continuously increase after the Zn doping and Pd modification, suggesting the positive effect of Zn and Pd on charge separation.

Table S3. Fluorescence lifetimes related to the TS-PL spectra of WO_3, $Zn_{0.35}$-WO_3 and $Pd_5/Zn_{0.35}$-WO_3.

	A_1	τ_1/ns	A_2	τ_2/ns	τ/ns
WO_3	634.68	2.53	478.78	2.56	2.54
$Zn_{0.35}$-WO_3	489.67	2.29	297.36	2.27	2.28
$Pd_5/Zn_{0.35}$-WO_3	173.70	1.85	258.97	1.87	1.86

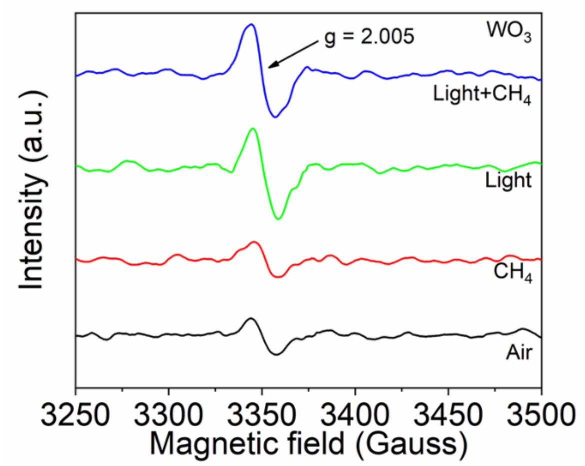

Figure S10. In-situ EPR signals of WO_3 collected under different conditions.

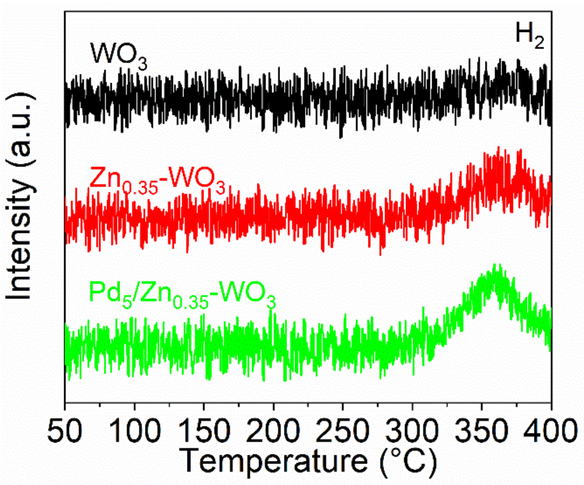

Figure S11. TPD-MS profiles of H_2 over WO_3, $Zn_{0.35}$-WO_3 and $Pd_5/Zn_{0.35}$-WO_3.

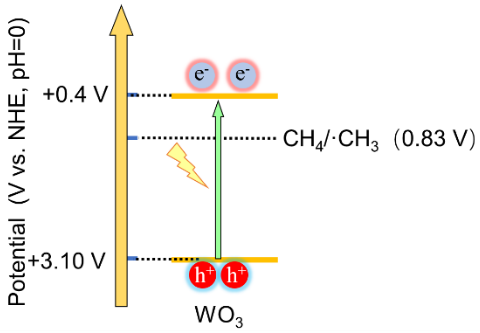

Figure S12. Illustration of the band structures of WO_3 and the redox potentials for oxidizing CH_4 to $\cdot CH_3$.

In addition, the valence band maximum of WO_3 (3.10 V vs. NHE) has a lower potential than the potential for oxidizing CH_4 to $\cdot CH_3$ (0.83 V vs. NHE), suggesting that the photogenerated holes are thermodynamically feasible for driving the oxidation of CH_4 to produce methyl radicals.

References

[S1] J. Song, Z. F. Huang, L. Pan et al., "Oxygen-deficient tungsten oxide as versatile and efficient hydrogenation catalyst," *ACS Catalysis*, vol. 5, pp. 6594-6599, 2015.

[S2] N. Zhang, A. Jalil, D. Wu et al., "Refining defect states in $W_{18}O_{49}$ by Mo doping: a strategy for tuning N_2 activation towards solar-driven nitrogen fixation," *Journal of the American Chemical Society*, vol. 140, pp. 9434-9443, 2018.

[S3] L. Meng, Z. Chen, Z. Ma et al., "Gold plasmon-induced photocatalytic dehydrogenative coupling of methane to ethane on polar oxide surfaces," *Energy & Environmental Science*, vol. 11, pp. 294-298, 2018.

[S4] S. Wu, X. Tan, J. Lei et al., "Ga-doped and Pt-loaded porous TiO_2–SiO_2 for photocatalytic nonoxidative coupling of methane," *Journal of the American Chemical Society*, vol. 141, pp. 6592-6600, 2019.

[S5] Z. Y. Chen, S. Q. Wu, J. Y. Ma et al., "Non-oxidative coupling of methane: N-type doping of niobium single atoms in TiO_2-SiO_2 induces electron localization," *Angewandte Chemie International Edition*, vol. 60, pp. 11901-11909, 2021.

[S6] W. B. Jiang, J. X. Low, K. K. Mao et al., "Pd-modified ZnO-Au enabling alkoxy intermediates formation and dehydrogenation for photocatalytic conversion of methane to ethylene," *Journal of the American Chemical Society*, vol. 143, pp. 269-278, 2021.

[S7] X. Yu, V. D. Waele, A. Löfberg et al., "Selective photocatalytic conversion of methane into carbon monoxide over Zinc-heteropolyacid-titania nanocomposites," *Nature Communication*, vol. 10, pp. 700, 2019.

[S8] X. Yu, V. L. Zholobenko, S. Moldovan et al., "Stoichiometric methane conversion to ethane using photochemical looping at ambient temperature," *Nature Energy*, vol. 5, pp. 511-519, 2020.

[S9] X. Li, J. Xie, H. Rao et al., "Platinum- and CuO_x-decorated TiO_2 photocatalyst for oxidative coupling of methane to C_2 hydrocarbons in a flow

reactor," *Angewandte Chemie International Edition*, vol. 59, pp. 19702-19707, 2020.

[S10] L. Yu, Y. Shao, D. Li, "Direct combination of hydrogen evolution from water and methane conversion in a photocatalytic system over Pt/TiO_2," *Applied Catalysis B: Environmental*, vol. 204, pp. 216-223, 2017.

[S11] J. Y. Ma, X. J. Tan, Q. Q. Zhang et al., "Exploring the size effect of Pt nanoparticles on the photocatalytic nonoxidative coupling of methane," *ACS Catalysis*, vol. 11, pp. 3352-3360, 2021.

[S12] S. P. Singh, A. Yamamoto, E. Fudo et al., "A Pd-Bi dual-cocatalyst-loaded gallium oxide photocatalyst for selective and stable nonoxidative coupling of methane," *ACS Catalysis*, vol. 11, pp. 13768-13781, 2021.
